# Supplementary material for: Comparative Proteomics of Root Apex and Root Elongation Zones Provides Insights into Molecular Mechanisms for Drought Stress and Recovery Adjustment in Switchgrass
Source: Proteomes. 2020 Feb 19;8(1):3. doi: 10.3390/proteomes8010003 (PMC7151713; doi:10.3390/proteomes8010003)
Supplement: Supplementary file 1 [file proteomes-08-00003-s001.zip › Final_Revised_Supplemental Data/Supplementary Table S1 Pooling strategy for fractions.pdf]

Supplementary Table S2 Pooling strategy for fractions 1-48 from 1st dimension separation

First Dimension Fraction #			New Second Dimension Sample #
1-4 to one tube		pooled →	Too low A_{214}, not analyzed
5	27	pooled →	1
6	28	pooled →	2
7	29	pooled →	3
8	30	pooled →	4
9	31	pooled →	5
10	32	pooled →	6
11	33	pooled →	7
12	34	pooled →	8
13	35	pooled →	9
14	36	pooled →	10
15	37	pooled →	11
16	38	pooled →	12
17	39	pooled →	13
18	40	pooled →	14
19	41	pooled →	15
20	42	pooled →	16
21	43	pooled →	17
22	44	pooled →	18
23	45	pooled →	19
24	46	pooled →	20
25	47	pooled →	21
26	48	pooled →	22
